# TUSC2 immunogene enhances efficacy of chemo-immuno combination on KRAS/LKB1 mutant NSCLC in humanized mouse model

**DOI:** 10.1038/s42003-022-03103-7

**Published:** 2022-02-24

**Authors:** Ismail M. Meraz, Mourad Majidi, RuPing Shao, Feng Meng, Min Jin Ha, Elizabeth Shpall, Jack A. Roth

**Affiliations:** 1 Department of Thoracic and Cardiovascular Surgery, The University of Texas MD Anderson Cancer Center, Houston, TX USA; 2 Department of Biostatistics, Graduate School of Public Health, Yonsei University, Seoul, Korea; 3 Department of Stem Cell Transplantation, The University of Texas MD Anderson Cancer Center, Houston, TX USA; 4 Department of Thoracic Medical Oncology, The University of Texas MD Anderson Cancer Center, Houston, TX USA

**Keywords:** Non-small-cell lung cancer, Cancer immunotherapy

## Abstract

KRAS/LKB1 (STK11) NSCLC metastatic tumors are intrinsically resistant to anti-PD-1 or PD-L1 immunotherapy. In this study, we use a humanized mouse model to show that while carboplatin plus pembrolizumab reduce tumor growth moderately and transiently, the addition of the tumor suppressor gene TUSC2, delivered systemically in nanovesicles, to this combination, eradicates tumors in the majority of animals. Immunoprofiling of the tumor microenvironment shows the addition of TUSC2 mediates: (a) significant infiltration of reconstituted human functional cytotoxic T cells, natural killer cells, and dendritic cells; (b) induction of antigen-specific T cell responses; (c) enrichment of functional central and memory effector T cells; and (d) decreased levels of PD-1^+^ T cells, myeloid-derived suppressor cells, Tregs, and M2 tumor associated macrophages. Depletion studies show the presence of functional central and memory effector T cells are required for the efficacy. TUSC2 sensitizes KRAS/LKB1 tumors to carboplatin plus pembrolizumab through modulation of the immune contexture towards a pro-immune tumor microenvironment.

## Abstract (web summary)

Meraz et al. explore the antitumor efficacy of TUSC2 tumor suppressor genetherapy via nanovisicles in combination with carboplatin and pembrolizumab against KRAS-LKB1 mutant NSCLC in humanized mouse model. They demonstrate a robust response and perform immune profiling studies, which show the development of a cytotoxic T cell effector response and effector memory cells.

## Introduction

Approximately half of NSCLC patients with activating KRAS mutations have deletions or inactivating mutations in the serine/threonine kinase 11 gene, STK11 (LKB1)^1,2^. The co-occurrence of KRAS mutation and LKB1 loss (KL) is associated with poor prognosis, due to an increase in metastatic burden and resistance to therapy^1,3,4^. Cytotoxic chemotherapy was the standard of care for the majority of patients diagnosed with metastatic NSCLC, irrespective of the histological subtype until the recent approval of immune checkpoint blockade therapy (ICB)^5^. Currently, chemotherapy plus ICB or ICB alone (if PD-L1 is >50) has been used as a first-line of treatment for metastatic NSCLC. Carboplatin with pemetrexed/Nab-Paclitaxel combined with pembrolizumab/Atezolizumab improved overall survival (OS) relative to chemotherapy alone in both adenocarcinoma and squamous cancers^6,7^. Monoimmunotherapy only shows clinical benefit among patients with higher programmed death protein ligand 1 (PD-L1) expression and with no oncogenic driver mutations such as EGFR, ALK, or ROS1. Worse OS and progression-free survival PFS (PFS) outcomes were found in patients with KL subtypes receiving chemotherapy and immunotherapy alone, or in combination, as compared with their wild-type groups^4^.

TUSC2, a tumor suppression gene, is completely absent or weakly expressed in the majority of NSCLC. TUSC2 is multifactorial in its mechanism of action and regulates a wide array of cellular processes, including apoptosis induction through broad multikinase inhibition and immune-system stimulation. We have recently established the role of TUSC2 in activating innate and adaptive immunity. TUSC2 selectively augments natural killer (NK) cells and cytotoxic T lymphocyte (CTL) activity in peripheral blood and in the tumor microenvironment and induces a Th1-mediated immune response, as well as downregulates myeloid-derived suppressor cells (MDSC) and regulatory T cells (Treg)^8^. We also showed that TUSC2 restoration downregulates PD-L1 expression in NSCLC and synergizes with anti-PD-1 in syngeneic KRAS mutant lung cancer mouse models^8,9^. A phase I study showed the safety of nanovesicle mediated-TUSC2 delivery^10^. TUSC2 nanovesicles work synergistically with tyrosine kinase inhibitors (TKIs), such as EGFR and AKT inhibitors, erlotinib, and M2206, respectively, through downregulating the oncogenic PI3K/Akt/mTOR pathway^11,12^. Inhibition of the latter was shown to induce expansion of tumor infiltrated lymphocytes (TILs), promoting a memory T cell phenotype^13^. Currently, an opened clinical trial evaluating TUSC2 synergy with a third TKI generation, osimertinib, is recruiting NSCLC patients (ClinicalTrials.gov Identifier: NCT04486833).

We recently developed an improved humanized mouse model using fresh human umbilical cord blood derived CD34^+^ stem cells^14^. The reconstituted humanized mice have a fully competent human immune system with major functional immune populations, which showed antigen-specific T cell responses as well as antitumor activity with immune checkpoint blockade therapy. This model provides a unique opportunity for establishing an effective drug screening workflow for immunotherapy.

Standard chemo–immunotherapy combination therapy is much less effective against NSCLC KL subtypes due to their impaired immunogenicity^1,4^. Silencing of STING signaling was found to be associated with immune evasion in KL cells, which might impair innate activation and antigen presentation^15^. In this study, we tested the hypothesis that modulation of the tumor immune microenvironment by TUSC2 immunogene therapy, which induces apoptosis in tumor cells and promotes a variety of innate and adaptive immune responses such as release of tumor-associated antigen, antigen processing and presentation, CTL responses, would alter the outcome of chemo–immunotherapy combination in a humanized mouse model implanted with KL A549 xenografts. A robust antitumor activity was found, which was significantly superior to a standard chemo–immunotherapy combination. The efficacy was associated with induction of an antitumor immune response through generating functionally active central and effector memory T cells.

## Results

### Carboplatin plus pembrolizumab induces transient antitumor activity

**Fig. 1:**
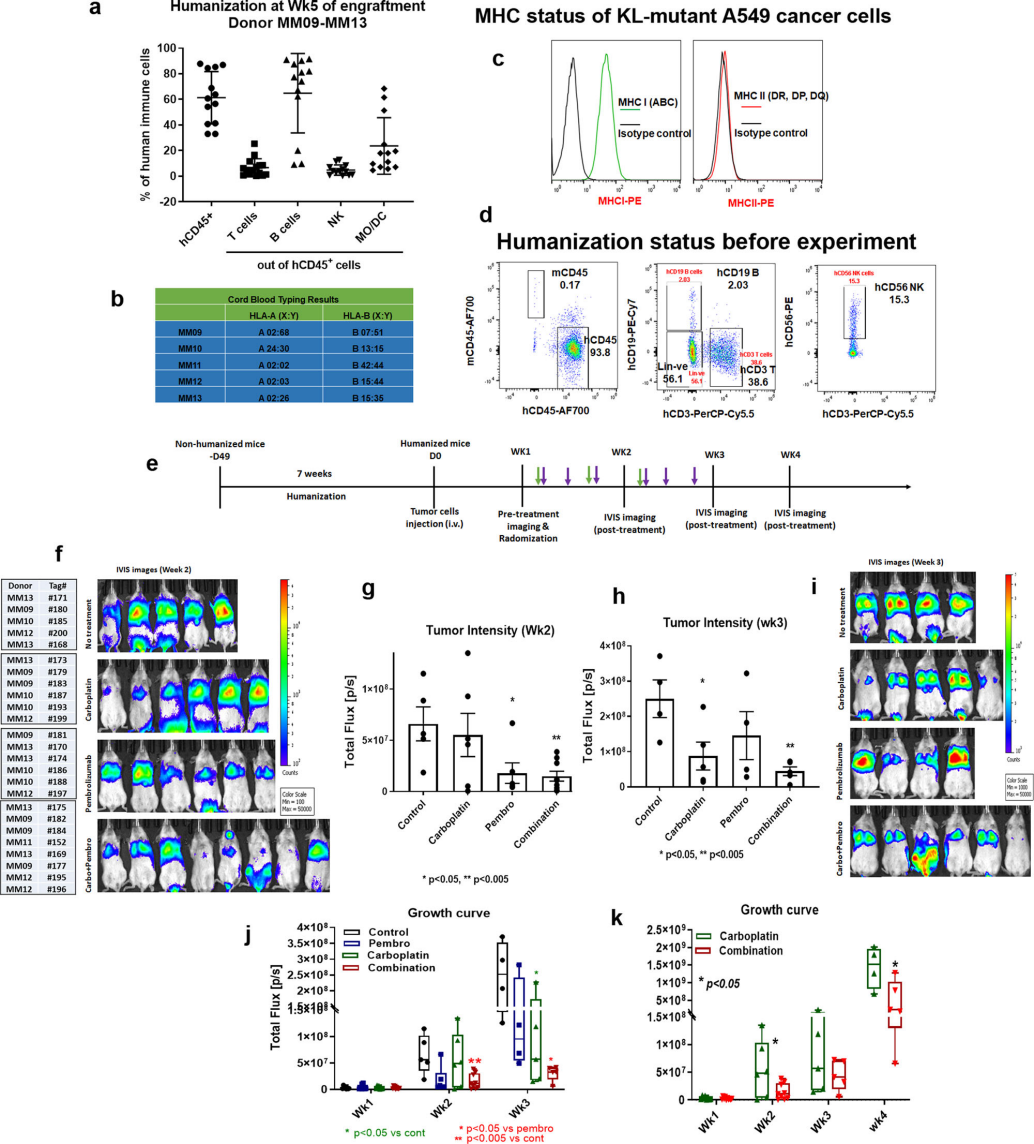
Effect of chemo–immunotherapy combination on KRAS/LKB1 mutant lung metastasis in humanized mice. **a** NSG mice were humanized through human CD34^+^ stem cells implantation into irradiated mice and the level of reconstitution of human immune cells into mice system after 5 weeks of post-humanization is shown. **b** Human HLA typing results of both HLA-A and HLA-B locus of umbilical cord blood donors. **c** Expression level of MHC class I and II on KRAS/LKB1 mutant cancer cell line. The level of expression of MHC I and MHC II on A549 was determined based on the expression level on PBMCs, which is used as a positive control in the experiment. **d** Scatter plot analysis to show the level of major human immune cells in humanized mice. A hundred microliters of blood collected from tail vein and immune cells were stained and analyzed by flow cytometry. **e** Treatment strategy where 7–8 weeks post humanized mice were implanted with tumor and lung metastases were treated with carboplatin (10 mg/kg) every other day (purple arrow) for 2 weeks and pembrolizumab (250 ug/mouse) every 3–4 days for 3X (green arrow). **f** Small animal imaging was done by IVIS 200 to determine the bioluminescence signal from tumor cells at week 2. **g**, **h** Quantitative analysis of tumor intensity determined from IVIS imaging at week 2 and week 3 respectively. **i** IVIS imaging of tumor-bearing humanized mice in different treatment groups at week 3. **j** Tumor growth curve shows the growth of lung metastasis and the response to the treatment at different time points. **k** Tumor growth curve comparison between carboplatin alone and carboplatin + pembrolizumab combination treatment groups at extended time points. The results shown are from one of three independent experiments with similar results; bars, SD. *P < 0.05, **P < 0.005, ***P < 0.0005.

Humanized mice implanted with KL A549 xenografts were treated with carboplatin combined with pembrolizumab to assess the antitumor efficacy of this model. Since KL NSCLC patients are not responsive to this combination, a similar result was predicted in this model. Fresh CD34 stem cell derived humanized mice developed from different cord blood donors were randomized into different experimental arms. Figure 1a shows the average percentage levels of human reconstituted CD45^+^ T, B, NK, and DC cells in mice, 5 weeks post CD34 engraftment, as characterized by multicolor flow cytometry. Cord blood from different donors were typed for the most common HLA-A and HLA-B loci (Fig. 1b). A549 cells exhibited low expression of MHC I and no expression of MHC II (Fig. 1c). The representative scatter plot shows human immune cell reconstitution status immediately before treatment was initiated.

Mice were repopulated with over 90% human CD45^+^ leukocytes, about 30% T lymphocytes, and 10–15% NK cells (Fig. 1d) after initial engraftment. Lung metastases were initiated by intravenous injection of KL mutant A549 cells. Mice were treated with pembrolizumab (250 μg/mouse) and carboplatin (10 mg/kg) according to the treatment strategy shown in Fig. 1e. Two weeks posttreatment, pembrolizumab monotherapy and carboplatin plus pembrolizumab combination, had similar antitumor efficacy (*P < 0.05; **P < 0.005) (Fig. 1g). Tumors were reduced significantly when compared to control and carboplatin alone. At week 3, efficacy of the combination was superior to that of carboplatin or pembrolizumab monotherapies (**P < 0.005) (Fig. 1h). Pembrolizumab efficacy was diminished at week 3, whereas carboplatin did not reduce tumor growth until week 3, *P < 0.05 (Fig. 1f–i). After the third week, tumors were no longer responsive to the combination or to both single agents, and continued progressive growth (Fig. 1j, k). These results validate this model as similar response trends occurred in KL NSCLC patients treated with carboplatin, pembrolizumab, or the combination of both with a non-durable response followed by tumor regrowth.

### TUSC2 synergizes with pembrolizumab

**Fig. 2:**
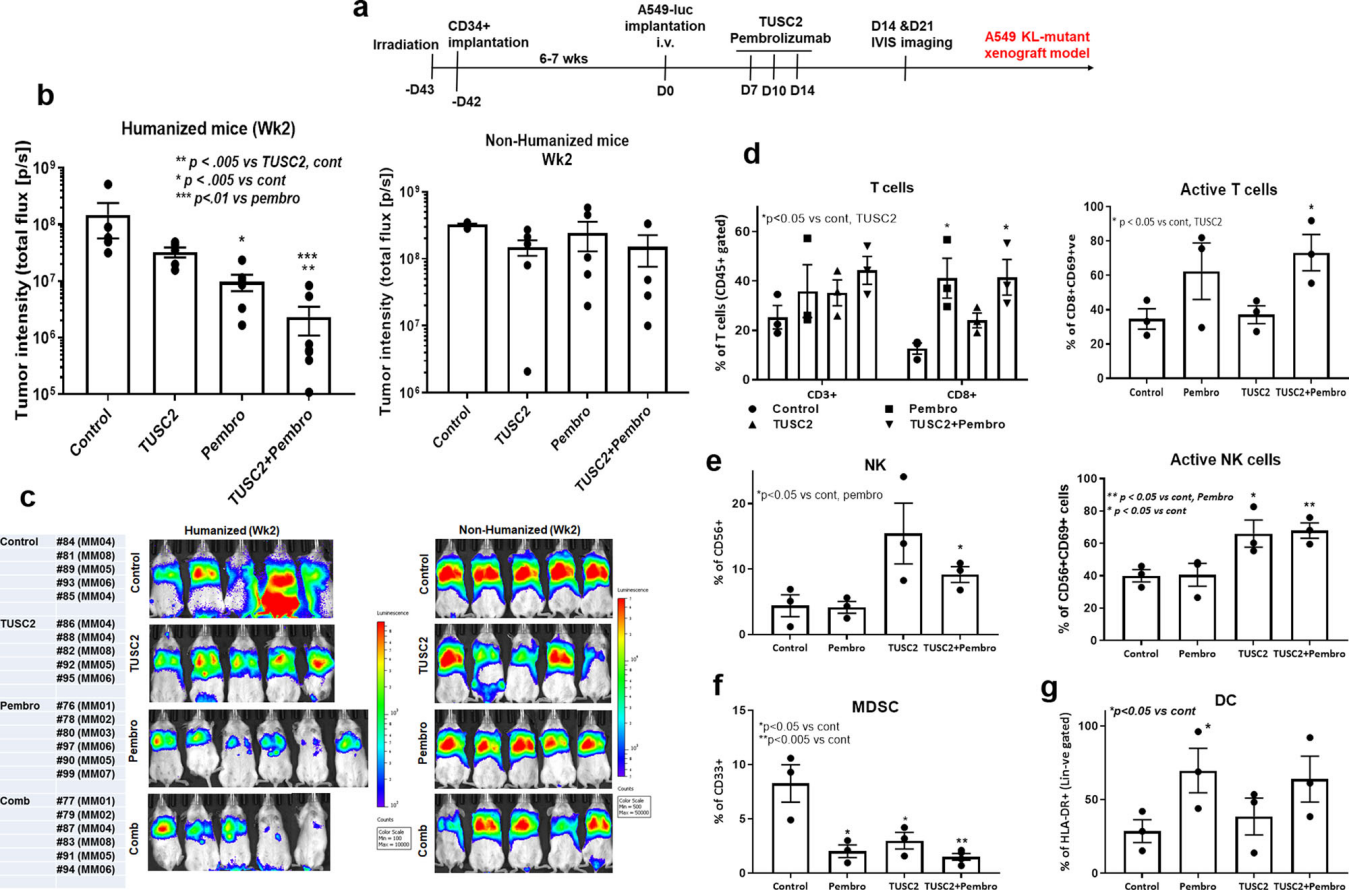
Synergistic antitumor effect of TUSC2 immunogene therapy with pembrolizumab on KRAS/LKB1 lung metastases in the humanized mouse model. **a** Treatment strategy shows that tumors were implanted into 6–7 weeks post humanized mice and lung metastasis were treated with TUSC2 injection through i.v. (25 ug/mouse) on every 3 days for 3X and pembrolizumab (250 ug/mouse) on every 3–4 days for 3X. **b** Tumor-bearing humanized mice were images by IVIS 200 and tumor intensity was measured and quantitated and level of tumor reduction by treatment was shown in both humanized and non-humanized mouse models. **c** bioluminescence imaging of performed by IVIS 200 to visualize the intensity of tumor burden on mice in different treatment groups both in humanized mice and non-humanized mouse system. This experiment was repeated three times with 7–8 mice/group used in each experiment, bars, SD. *P < 0.05, **P < 0.005, ***P < 0.0005. **d** Tumors were analyzed through single cells preparation and stained for human T and activated T cells in tumor microenvironment. **e** infiltrating NK and activating NK cells were analyzed in tumors. **f**, **g** Level of MDSC and HLA-DR + DC in tumor were determined through flow cytometry analysis of tumor tissues. Data are shown as mean percentage ± SD, *n* = 5. *P < 0.05, **P < 0.005, ***P < 0.0005.

We have previously shown that the combination of TUSC2 and anti-PD1 inhibited tumor growth synergistically in subcutaneous and metastatic NSCLC KRAS mutant syngeneic mouse models (8). To determine whether this synergy also applies to the KL subtype, humanized mice harboring KL A549 lung metastases were treated with TUSC2, pembrolizumab, or the combination. The TUSC2 gene was packaged in DOTAP-cholesterol(DC) nanovesicles for intravenous systemic delivery (at a dose of 25 μg of plasmid DNA, 10 nmol DC in 100 μL of 5% dextrose in water every 48 h for a total of three injections). The treatment strategies are shown in Fig. 2a. Both TUSC2 and pembrolizumab monotherapies reduced the tumor burden significantly, *P < 0.05, although pembrolizumab was moderately more effective. TUSC2 plus pembrolizumab inhibited tumor growth synergistically, ***P < 0.0005 (Fig. 2b, c). There was no antitumor effect of pembrolizumab in non-humanized mice. TUSC2 also showed synergistic antitumor activity with nivolumab in the same model (Supplement Fig. 1), highlighting the role of TUSC2 in rendering KL tumors more sensitive to immune checkpoint blockade.

To identify the immunological features associated with efficacy of this combination, we performed in-depth immune profiling of the tumor microenvironment. An increased number of reconstituted human CD3^+^ T cells was found in all groups, compared with the untreated control. CD8^+^ T cells were significantly upregulated (*P < 0.05) by pembrolizumab and its combination with TUSC2 (Fig. 2d). Levels of activated CD8^+^ T cells (CD8^+^CD69^+^) were also significantly increased in the combination group (*P < 0.05), and were slightly higher than the pembrolizumab group (Fig. 2d). There was no effect of pembrolizumab on NK/activated NK cells, whereas TUSC2 alone enhanced their levels significantly (*P < 0.05), indicating TUSC2 regulation of NK activation, which is consistent with our previous finding reported in syngeneic mice^8^. The combination had a similar effect as TUSC2 monotherapy (Fig. 2e). TUSC2, pembrolizumab, and the combination, were all associated with significant decrease of reconstituted human MDSCs (CD33^+^ ve), *P < 0.05, **P < 0.005 (Fig. 2f). Pembrolizumab and the combination had a profound stimulatory effect on HLA-DR^+^ DCs, *P < 0.05 (Fig. 2g). TUSC2 restored expression alone enhanced HLA-DR^+^ DC levels moderately. Taken together, the result shows that the combination of TUSC2 and pembrolizumab inhibited tumor growth synergistically, a response that was significantly more effective and durable than that of carboplatin plus pembrolizumab.

### TUSC2 plus carboplatin and pembrolizumab combination eradicates KL tumors

**Fig. 3:**
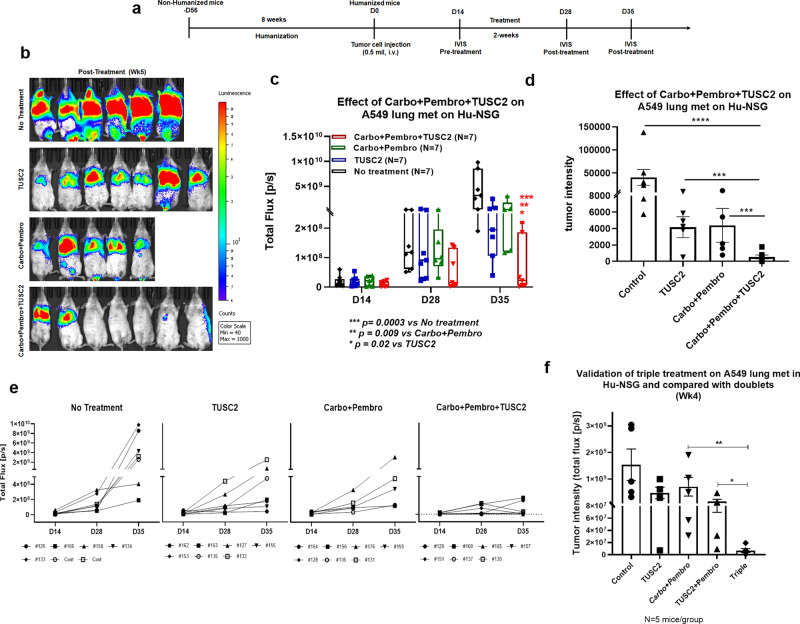
The TUSC2 immunogene enhances the chemo–immunotherapy combination on KRAS/LKB1 mutant lung metastases in humanized mouse. **a** Carboplatin, pembrolizumab, and TUSC2 triple combination treatment strategy where 7–8 weeks post-humanized mice were implanted with KRAS/LKB1 tumors. After 2 weeks, mice were imaged to determine the level of lung metastases, L and mice were randomized. Lung metastases were treated with carboplatin (10 mg/kg) on every other day for 2 weeks, pembrolizumab (250 ug/mouse) on every 3–4 days for 3X, and TUSC2 (25 ug/mouse) on every 3 days for 3X. Mice were imaged at different time points. **b** Mice were imaged by IVIS 200 for bioluminescence signals coming from tumor cells and tumor burden is shown in different treatment groups. **c** Tumor intensity was measured through bioluminescence imaging. The growth curve shows the progression of tumor growth among different treatment groups. **d** Rate of tumor growth was determined by percentage change in tumor intensity between pre- (week 2) and posttreatment (week 5). **e** Individual mouse response curves are shown in each treatment group to understand the treatment effect on each humanized mouse. **f** Validation of antitumor activity of triple agent treatment was determined in separate humanized mouse experiments where doublets such as carboplatin + pembrolizumab and TUSC2 + pembrolizumab combinations were compared side by side. The humanized mouse experiment with triple agent treatment was one of three independent experiments using 7–8 humanized mice/group. bars, SD. Statistics were shown at a significance level of *p* < 0.05 unless otherwise noted. *P < 0.05, **P < 0.005, ***P < 0.0005.

TUSC2 was then added to the carboplatin and pembrolizumab combination. NSG mouse humanization and treatment protocol strategies are shown in Fig. 3a. TUSC2 plus pembrolizumab and carboplatin plus pembrolizumab dual combinations were used as controls. We found that TUSC2 inhibited tumor growth to the same extent as carboplatin plus pembrolizumab (Fig. 3b–d). TUSC2 combination with pembrolizumab and carboplatin eradicated tumors completely in some mice, *P < 0.05. Bioluminescence imaging showed five out of seven humanized mice with no tumor burden at week 5 (Fig. 3b). The other two less responsive tumors did exhibit more growth inhibition than those in the carboplatin plus pembrolizumab group. The percentage change in tumor intensity between weeks 2 and five showed that the triple agent treatment significantly reduced or eradicated tumors as compared with the chemo–immunotherapy combination, Fig. 3d (***P < 0005). Individual growth curves showed that all mice in the triple treatment group had little or no tumor signal in lung (Fig. 3e). Complete tumor eradication was validated in several independent experiments (Fig. 3f, supplement Fig. 2.). The results clearly demonstrate that restoration of TUSC2 expression profoundly enhances KL tumor response to conventional chemo–immunotherapy. This triple combination also induced strong antitumor immune responses in a highly aggressive aPD1 resistant KRAS mutant (G12V) CMT167 syngeneic mouse model (supplement Fig. 5).

### Efficacy of TUSC2 plus carboplatin and pembrolizumab combination is associated with an increase in immune-stimulatory cells within the TME

**Fig. 4:**
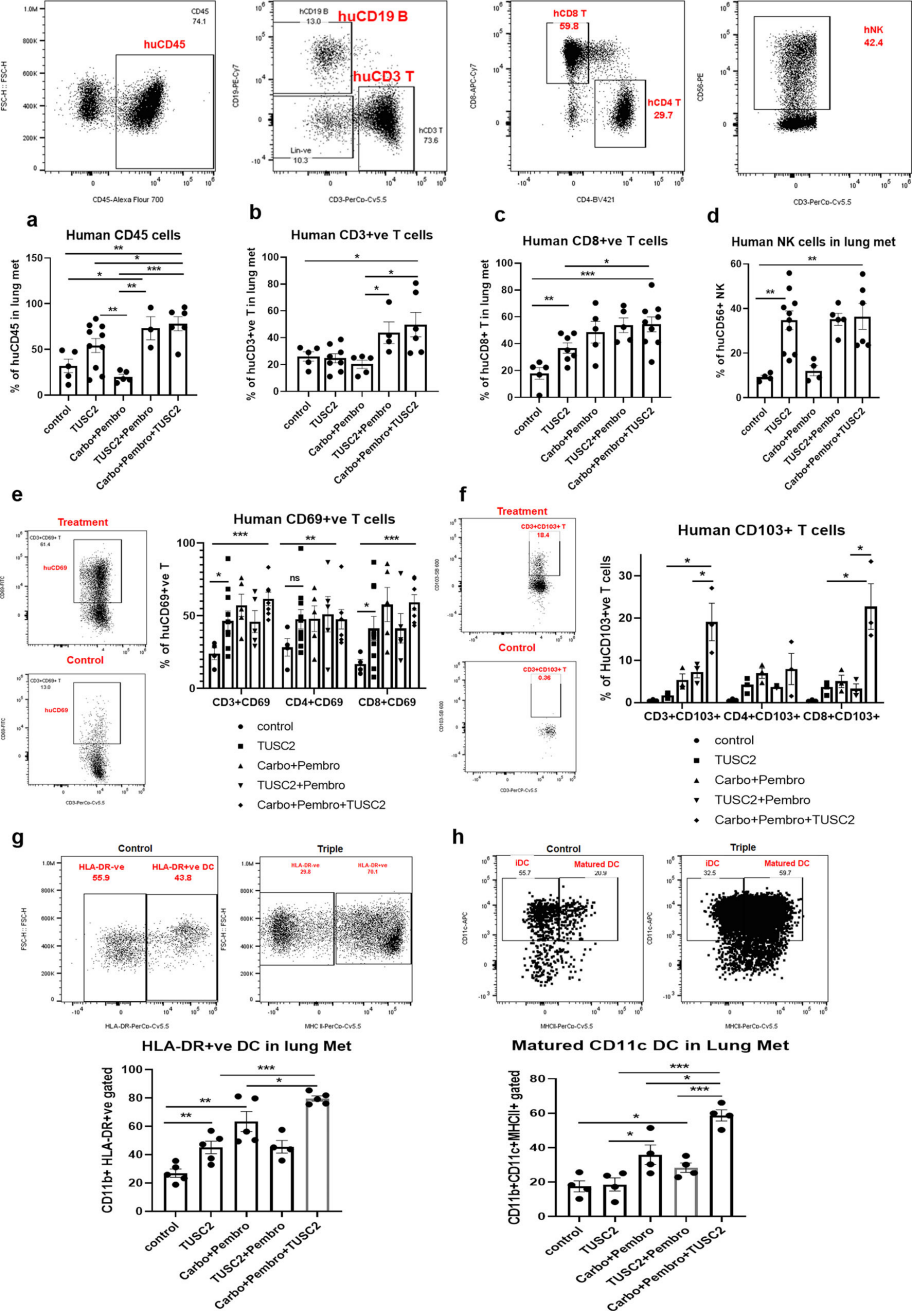
Antitumor immune response mediated by carboplatin + pembrolizumab + TUSC2 combination on KRAS/LKB1 lung metastases in the humanized mouse model. **a**–**d** Lung metastases tissues were analyzed for infiltrating human immune cells. Single-cell suspensions were prepared from fresh lung metastases and in-depth immune analyses were performed using flow cytometry for determining human CD45+ leukocytes, CD3+ T, CD8+ T, and NK, cells. The level of human immune cells are shown for different treatment groups. **e** Level of activating CD3+ T, CD4+ T, and CD8+ T cells were determined by the expression of CD69 expressing markers on infiltrating T cells among different treatment groups. **f** Percentages of tissue-resident T cells (T_RM_) in tumors and their alteration by triple agent treatment. CD103+ expressing T cells were considered as T_RM_. **g** Level of HLA-DR + DC cells was determined among the lineage-negative population. **h** Matured CD11c positive DC were determined based on subsequent gating on CD11b+ > CD11c+ > HLA-DR+ and the level of alteration of CD11c DC were shown under different treatments. Statistics are shown at a significance level of *p* < 0.05 unless otherwise noted. Data are shown as mean percentage ± SD, *n* = 5. *P* < 0.05, **P < 0.005, ***P < 0.0005.

To characterize the immunological impact of this triple agent combination, we profiled major reconstituted human immune populations in the tumor microenvironment in humanized mice by flow cytometry. Immunological features of the triple agent combination were compared with those of the dual treatments. As Fig. 4a–h show, the triple combination was associated with a significant increase number of reconstituted human (a) CD45^+^ (**P < 0005 vs control; *P < 0.05 vs TUSC2; ***P < 0.0005 vs carboplatin + pembrolizumab); (b) CD3^+^ T (*P < 0.05 vs control, *P < 0.05 vs carboplatin + pembrolizumab); (c) CD8^+^ T (***P < 0.0005 vs control, *P < 0.05 vs TUSC2); (d) NK effector cells (**P < 0.0005 vs control, *P < 0.05 vs carboplatin + pembrolizumab); (e) tumor infiltrated active T cells (CD3^+^CD69^+^, CD4^+^CD69^+^, CD8^+^CD69^+^); (f) tissue resident memory T cells (CD3^+^CD103^+^, CD4^+^CD103^+^, CD8^+^CD103^+^); and (g) dendritic cells, DC (HLA-DR^+^, CD86, CD40, and CD11c). Maturation on DCs by the triple agent combination is shown in supplement Fig. 3.

TUSC2 intravenous injection enhanced levels of CD45^+^ and CD8^+^ T cells significantly, although not to the extent of TUSC2 plus pembrolizumab or carboplatin plus pembrolizumab. TUSC2 had a profound stimulatory effect on the active NK cell population alone and when combined with other agents, validating NK stimulation by TUSC2, which we recently reported^8^. TUSC2 plus pembrolizumab was associated with better activation of CD45^+^ Tcells, CD3^+^ Tcell, CD8^+^ Tcell, and NK cells than carboplatin plus pembrolizumab. The data show that in the TME, the triple agent combination promotes a potent antitumor immunity, which is mediated largely by tumor infiltrating active lymphocytes, NK cells, and DC.

### Efficacy of TUSC2 plus carboplatin and pembrolizumab combination is associated with a significant decrease of immunosuppressive cells within the TME

**Fig. 5:**
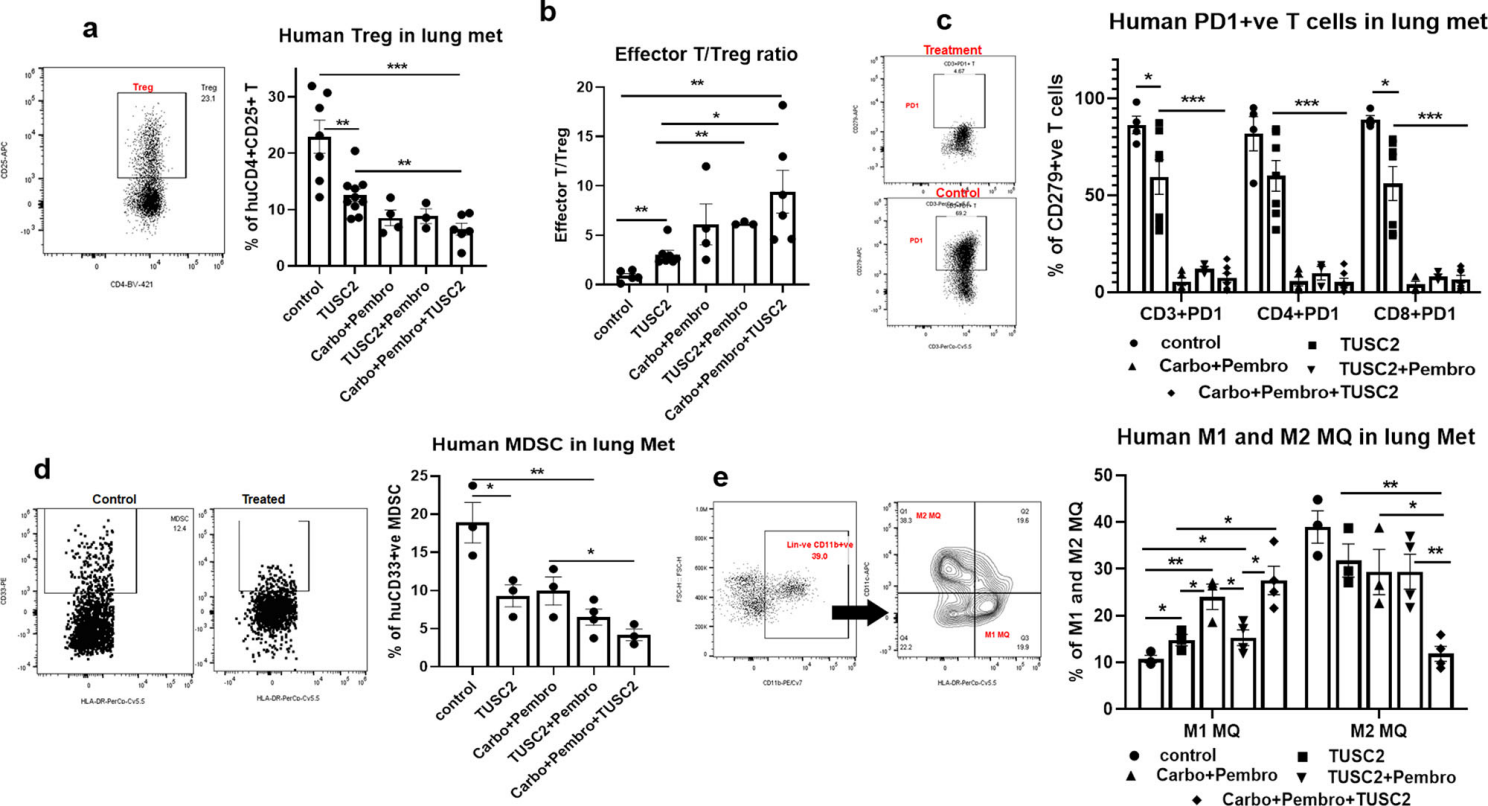
Effect of carboplatin + pembrolizumab + TUSC2 combination on human immune suppressive cells on KRAS/LKB1 lung metastases in the humanized mouse model. In-depth immune analysis was performed using multicolor flow-cytometry using lymphoid and myeloid panels which determined regulatory T cells (Treg), MDSC, and the level of the TAM population in lung metastases. **a** The level of human Treg cells are shown for different treatment groups, **b** Ratio of CD8+ T/regulatory T cells in tumors, **c** level of PD-1 expressing human T cells and inhibition of PD-1 expression on T cells by treatments in the tumor microenvironment in humanized mice, **d** level of human MDSC based on expression of CD33+ HLA-DR−ve population and the changes of MDSC in different treatment groups shown. **e** Tumor-associated macrophages (TAM) population were analyzed in lung metastasis. M1 and M2 macrophages are shown in different treatment groups. Lin-ve > CD11b+ > CD163+ > HLA-DR^−^ gating strategies were used for determining the M2 macrophages. Statistics are shown at a significance level of *p* < 0.05 unless otherwise noted. Data are shown as mean percentage ± SD, *n* = 5. *P < 0.05, **P < 0.005, ***P < 0.0005.

The triple combination was also associated with a significant decrease of reconstituted tumor-infiltrating human immunosuppressive cells. These include: (a) regulatory T cells (Treg) (Fig. 5a, b), as demonstrated by the higher effector/Treg ratio (**P < 0.005 vs control, *P < 0.05 vs TUSC2); (b) PD1^+^ T cells (CD3^+^CD274^+^ T, CD4^+^CD274^+^ T, CD8^+^CD274^+^ T) (Fig. 5c); (c) myeloid-derived suppressor cells (MDSC) (Fig. 5d); and (d) M2 tumor associated macrophages (TAMs), (Fig. 5e) These results indicate that the triple combination diminishes the immunosuppressive TME, mediated largely by tumor-infiltrating MDSCs, TAMs and Tregs, significantly more than TUSC2 plus pembrolizumab or carboplatin plus pembrolizumab.

### Efficacy of the TUSC2 plus carboplatin and pembrolizumab combination is associated with a significant increase of functionally active memory T cells

**Fig. 6:**
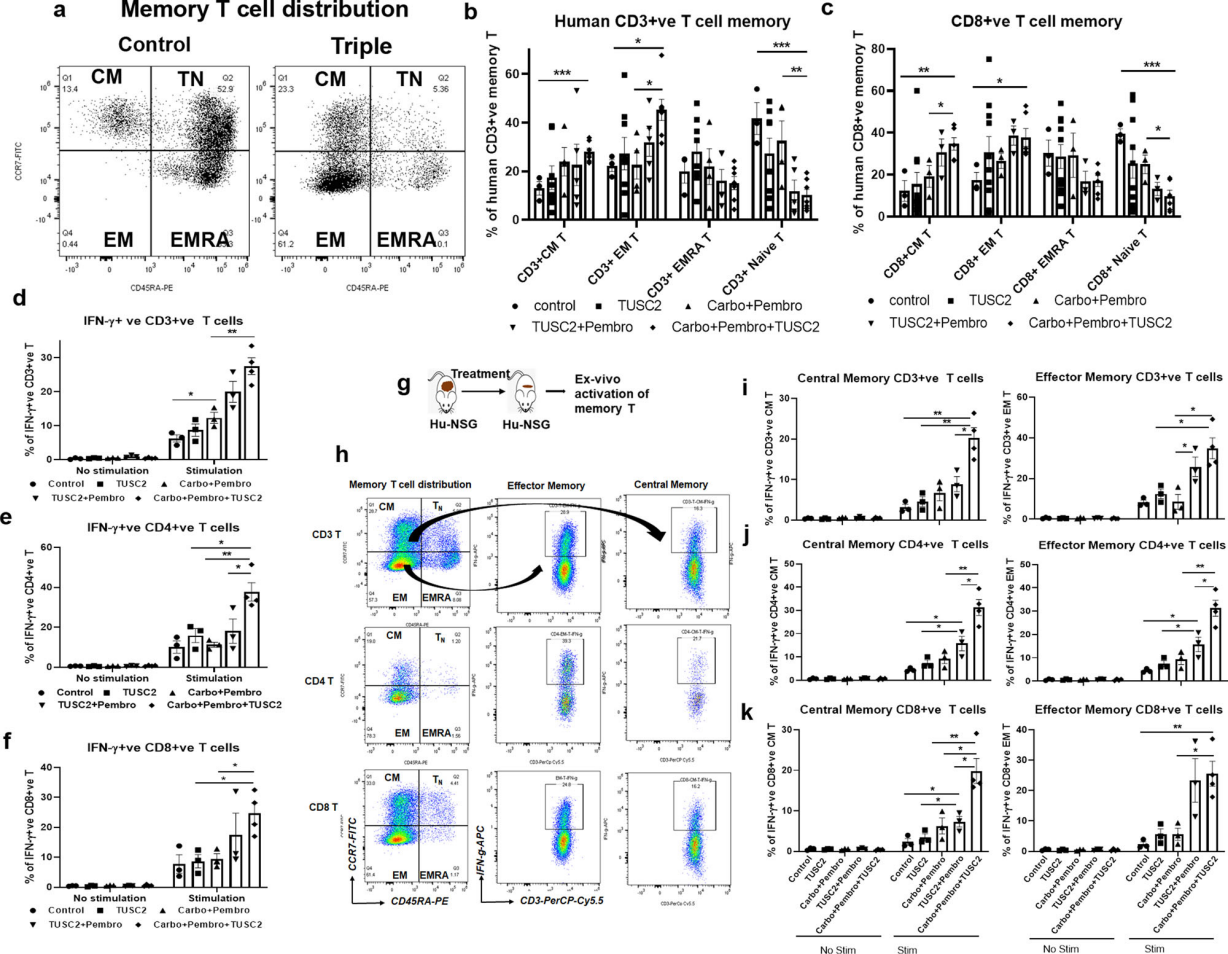
Distribution of human memory T cells and their ex-vivo activation in KRAS/LKB1 lung metastases in a humanized mouse model. In-depth immune analysis was performed using multicolor flow-cytometry using a memory T cell panel in lung metastases and ex-vivo activation of memory T cells was determined by IFN-γ intracellular staining. **a** Scatter plots show the memory T cell distribution in lung metastases in humanized mice. **b** Percentages of CD3+ ve central memory (CM), effector memory (EM), EMRA, and naïve T cells in the tumor microenvironment in tumor-bearing humanized mice treated with single, double, and triple agent combination treatments. **c** Percentages of CD8+ ve central memory (CM), effector memory (EM), EMRA, and naïve T cells in tumor microenvironment in tumor-bearing humanized mice treated with single, double, and triple agent combination treatments. **d**–**f** Tumor-bearing humanized derived T cells were stimulated in-vitro with PMA and Ionomycin and percentages of IFN-γ+ ve CD3+ T, CD4+ T, and CD8+ T were measured respectively. **g** Strategy of in-vitro activation of T cells. **h** Gating strategy for analyzing the activated CD3+, CD4+, and CD8+ effector and central memory T cells. **i**–**k** In-vitro activation of CD3+, CD4+, and CD8+ central and effector memory by determining percentages of IFN-γ+ ve T cells under stimulated and unstimulated conditions. Statistics are shown at a significance level of *p* < 0.05 unless otherwise noted. Data are shown as mean percentage ± SD, *n* = 5. *P < 0.05, **P < 0.005, ***P < 0.0005.

We evaluated the distribution and alteration of effector memory (EM) (CD3^+^/CD8^+^ CD45RA^−^ CCR7^−^), central memory (CM) (CD3^+^/CD8^+^ CD45RA^−^ CCR7^+^), and naïve (T_N_) (CD3^+^/CD8^+^ CD45RA^+^ CCR7^+^) T cells. CD3^+^ and CD8^+^ CM and EM populations were significantly increased following triple combination treatment compared with carboplatin plus pembrolizumab and TUSC2 plus pembrolizumab (CD3^+^CM, ***P < 0.0005; CD3^+^EM, *P < 0.05; CD8^+^CM, ***P < 0.0005; CD8^+^EM, *P < 0.05) (Fig. 6a–c). In contrast, both CD3^+^ and CD8^+^ Naïve T cells (T_N_) were significantly less prevalent in the triple agent treatment group, compared with the carboplatin plus pembrolizumab (CD3^+^ T_N_, **P < 0.005 vs carboplatin + pembrolizumab, ***P < 0.0005 vs control; CD8 + T_N_, *P < 0.05 vs carboplatin + pembrolizumab, ***P < 0.0005 vs control) (Fig. 6b, c).

In-vitro activation of T cells derived from tumors, and stimulated with PMA, resulted in a significant release of IFN-γ by CD3^+^, CD4^+^, and CD8^+^ T cells, regardless of the treatment group, indicating that these T cells are functionally active (Fig. 6d–f). Analysis of IFN-γ expression by tumor-infiltrating CD3^+^, CD4^+^, and CD8^+^ central and effector memory T cells revealed a significantly higher level in the triple combination (CD3^+^/CD4^+^ CM, **P < 0.005 vs carboplatin + pembrolizumab; CD8^+^ CM, *P < 0.05 vs carboplatin + pembrolizumab), (CD3^+^ EM,*P < 0.05, CD4^+^ EM, **P < 0.005, CD8^+^ EM,*P < 0.05) (Fig. 6i–k). TUSC2 plus pembrolizumab showed the second-largest increase in activating memory T cells, which was consistent with its antitumor activity shown in Fig. 3f. Thus, the triple agent combination yielded the highest number of functionally central and effector memory T cells in the tumor microenvironment.

### TUSC2+ carboplatin and pembrolizumab increased central and effector memory T cell-dependent antigen-specific responses

**Fig. 7:**
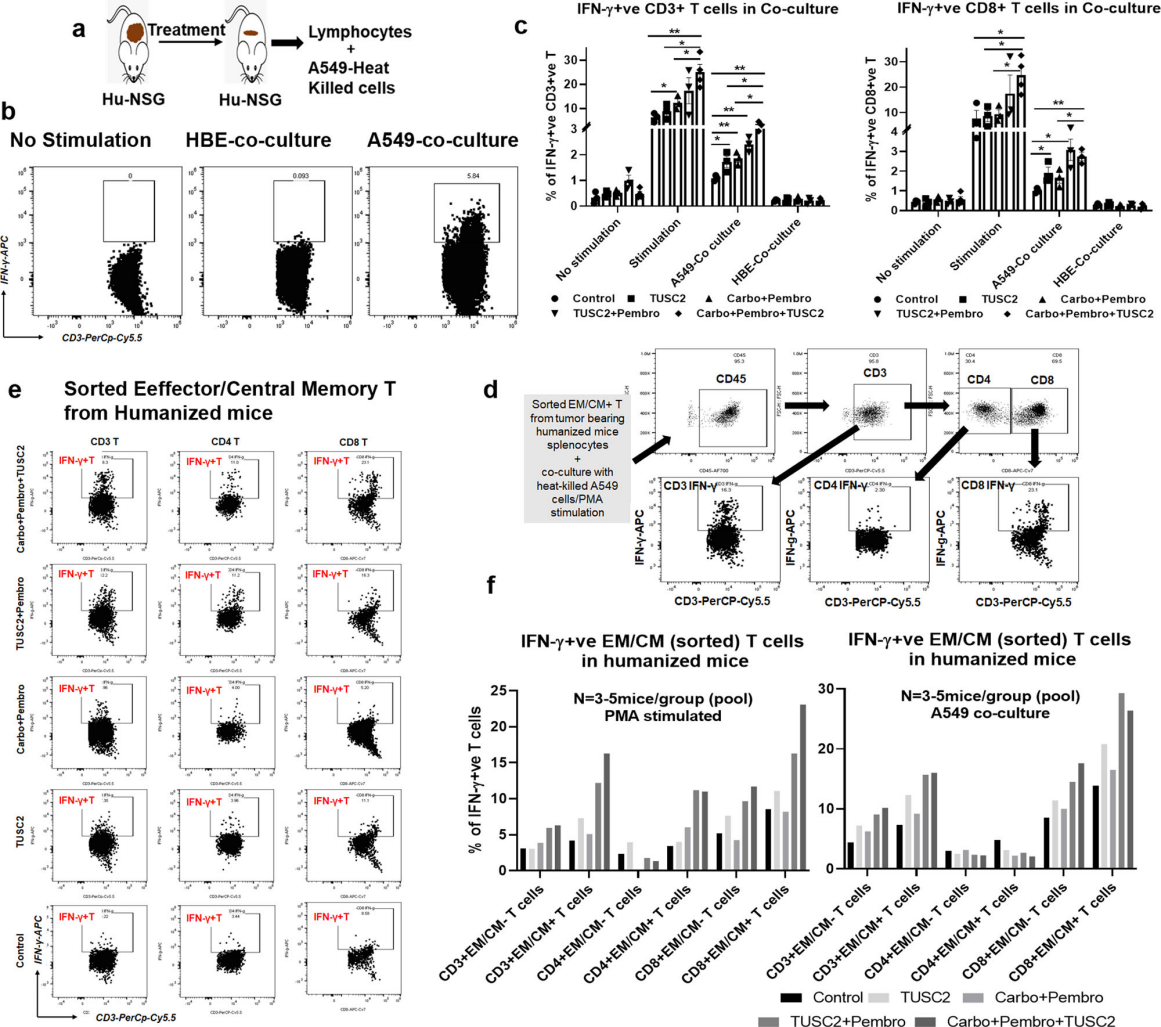
Memory T cell mediated antigen-specific T cell response in KRAS/LKB1 lung metastases in a humanized mouse model. In-depth immune analysis was performed using multicolor flow-cytometry using a memory T cell panel in lung metastases and ex-vivo activation of memory T cells were determined by IFN-γ intracellular staining. **a** Strategy shown for in-vitro activation of T cells by tumor antigens after harvesting from tumor bearing treated humanized mice. **b** Scatter plots show the gating strategy of IFN-g positive T cells after challenging with tumor antigen. **c** Antigen-specific CD3+ T and CD8+ T cell responses determined by measuring the percentages of IFN-g expression upon stimulation with tumor antigens. **d** Scatter plots showing the level of IFN-γ expressing CD3+, CD4+, and CD8+ sorted CM/EM+ T cells in different treatment groups **e** gating strategy of analysis of sorted central memory and effector memory T cells. **f** Level of IFN-g expressing sorted EM/CM+ T cells and EM/CM^−^ T cells after stimulation with either tumor antigens by co-culturing with heat-killed A549 cancer cells or PMA under different treatment conditions. Statistics were shown at a significance level of *p* < 0.05 unless otherwise noted. Data are shown as mean percentage ± SD, *n* = 5. *P < 0.05, **P < 0.005, ***P < 0.0005.

To assess antigen-specific T cells responses following treatment, T cells were harvested from tumor-bearing humanized mice and co-cultured with KL A549 cells, and intracellular IFN-γ expression was measured in vitro (Fig. 7a). Scatter plots show the gating of IFN-γ^+^ T cells for T cells in co-cultures without stimulation, with HLA matched human bronchial epithelial cells (HBE) and with A549 cancer cells (Fig. 7b). When T cells were co-cultured with A549 cells, an increased number of CD3^+^ IFN-γ^+^ T cells were found in all treatment groups compared to control, (*P < 0.05 vs TUSC2, **P < 0.005 vs carboplatin + pembrolizumab, **P < 0.005 vs TUSC2 + pembrolizumab) (Fig. 7c). However, increases of CD3^+^ IFN-γ^+^ and CD8^+^ IFN-γ^+^ T cells were the highest in the triple agent treatment group, *P < 0.05 (Fig. 7c). There was no IFN-γ^+^ T cell expression when T cells were not stimulated or co-cultured with HBE cells, indicating that the observed T cell responses with A549 co-culture were antigen-specific (Fig. 7c).

Finally, to determine whether the efficacy of TUSC2 plus carboplatin and pembrolizumab required effector (EM) and central memory (CM) T cells, we sorted and depleted EM/CM T cells from tumors, which were either stimulated with PMA or challenged with KL A549 cells in-vitro. The gating strategy used for the analysis of sorted EM/CM^+^ T cells is shown in Fig. 7d. The level of IFN-γ expression in sorted memory T cells is shown in scatter plots (Fig. 7e). The percentage of IFN-γ^+^ T cells was significantly higher in CD3^+^EM/CM^+^ T cells and CD8^+^EM/CM^+^ T cells than their respective depleted counterparts, CD3^+^EM/CM^−^ T and CD8^+^EM/CM^−^ (Fig. 7f). CD4^+^EM/CM^−^ T and CD4^+^EM/CM^+^ T cell had the lowest IFN-γ^+^ levels after stimulation with PMA or in co-culture with A549, suggesting that CD3^+^ and CD8^+^ EM/CM^+^ T cells are the main memory subsets for IFN-γ production. CD8^+^ EM/CM^+^ sorted T cells expressed high IFN-γ in every treatment group, although TUSC2 plus pembrolizumab and TUSC2+ carboplatin and pembrolizumab were the highest. Statistical analysis of IFN-γ responses from sorted Effector/Central memory T cells is shown in supplement Fig. 4. Taken together, the results show that the triple agent treatment induced strong antigen-specific T cells responses, which were greatly diminished when effector/central memory T cells were depleted.

## Discussion

Loss of LKB1 activity is prevalent in KRAS mutant lung adenocarcinoma and promotes treatment-resistant tumors and poor survival^2,4,16^. Patient populations with these abnormalities do not respond nor derive long-term survival benefits from any therapy, including cytotoxic, cytostatic, immunotherapies, or their combinations^1,17–22^. The reason behind the insufficient clinical utility for this subtype is impaired immunogenicity, rather than treatment potency and efficacy. We previously investigated the relationship between TUSC2 and LKB1 using multiple NSCLC cell lines with or without LKB1 expression^12^. TUSC2 showed selective sensitivity on LKB1-deficient cell lines as compared with wtLKB1 cell lines through upregulation of AMPK. In wtLKB1 expressing cells, LKB1 function is through direct activation of AMPK, thereby regulating apoptosis in response to energy stress^23^. In LKB1-deficient cells, there is no LKB1-AMPK interaction and TUSC2 transfection in LKB1-deficient cells stimulated AMPK phosphorylation at Th172, the same residue used by LKB1 proteins for coupling with AMPK, raising the possibility that TUSC2 and LKB1 might compete with each other for binding to the same functional protein-binding site on AMPK. The mechanism of immunosuppression by LKB mutation is not well studied. However, LKB1 downregulates STING signaling in the tumor microenvironment^15^, which suppresses innate immune activation and antigen presentation. We have shown previously and in the current study, that TUSC2 activates the innate immune response by inducing NK cell proliferation and Th1-mediated cytokine secretion. We also tested the therapeutic efficacy of TUSC2 in combination with the STING agonist (cGAMP) in the syngeneic CMT167 mouse model (KRAS mutant, aPD1 resistant) which showed enhanced antitumor activity, which was better than single-agent treatment. In this study, we have shown that TUSC2 immunogene therapy combined with carboplatin and pembrolizumab results in complete tumor eradication in over 65% of humanized mice implanted with KL NSCLC metastatic tumors. This impressive therapeutic benefit was reproducible in three blinded independent experiments. Tumor regression was also observed in the other fraction of animals but without complete eradication. This variation of tumor eradication might be explained by the variability of human immune reconstitution levels among humanized mice.

A very important consideration is the advanced model used. Many preclinical studies evaluating therapeutic strategies use models that are not highly predictive of success in clinical trials. Therefore, most drugs tested in current preclinical models fail in the clinic. To address this major limitation, we have developed an improved humanized mouse model, derived from fresh CD34^+^ stem cell engraftment in NOD.Cg-*Prkdc*^*scid*^ *Il2rg*^*tm1Wjl*^/SzJ (NSG) mice, without the onset of graft-versus-host disease (GVHD). We have demonstrated that this model replicates human tumor response to checkpoint blockade, mounts an immune response to tumor-associated antigens and not alloantigens, and is not stem cell donor-dependent for its immune response^14^. Although humanized mouse model is an advanced preclinical model useful for testing the immune cellular response and changes in the tumor microenvironment following treatment, there are limitations of this model. Biological variation is greater in humanized mice compared with syngeneic mice. The level of engraftment of CD34^+^ stem cells derived from different donors and the level of different immune subpopulations differentiated from stem cells are not homogeneous in humanized mice, although humanized mice generated from the same donor are relatively homogeneous in their engraftment. Another limitation is the HLA-compatibility match among humanized mice, which are generated from different donors with different HLA haplotypes. A complete match of HLA is difficult unless the system is autologous, although in our previous study, we showed that the antitumor effect of checkpoint blockade immunotherapy does not depend on HLA-status^14^.

The hypothesis behind designing this innovative triple agent combination was that restoration of the multifactorial tumor suppressor gene TUSC2, deficient in 80% of NSCLC, will modulate the tumor immune microenvironment by changing its context from an immunosuppressive to immunostimulatory milieu, which will prime tumors for better response to chemo–immunotherapy. TUSC2 regulates a wide range of cellular processes, including cell cycle arrest and apoptosis, through inhibition of several kinases and modulation of the tumor microenvironment (TME). TUSC2 activates diverse innate and adaptive immune responses and downregulates PD-1/PD-L1 signaling to enhance sensitivity to checkpoint blockade by nivolumab and pembrolizumab^8–12^.

For every randomized experiment, immunoprofiling analysis demonstrated high levels of reconstituted human immune cell populations in mice at different time points. Two weeks posttreatment, carboplatin plus pembrolizumab had similar antitumor activity as pembrolizumab did, which was significantly more effective than carboplatin alone. In week three, this dual combination was more effective than either single agent. However, this initial response, additive at best, was only transient and was followed by tumor regrowth. This model recapitulated the effect of carboplatin plus pembrolizumab reported in KL NSCLC patients^19,22^. Carboplatin plus pembrolizumab efficacy was significantly inferior to that of the TUSC2 plus pembrolizumab combination, which was synergistic and durable. However, with the latter, the tumor regressed substantially but was not completely eradicated. TUSC2 and nivolumab synergistic antitumor response were also shown in this model.

Correlation of immunological features of TME revealed that TUSC2 plus carboplatin and pembrolizumab altered the immune contexture towards a pro-immune tumor microenvironment, which was not observed post carboplatin plus pembrolizumab treatment. There was a shift from a pre-existing immune response to a therapy-induced immune response. Reconstituted human CTLs, activated T cells, NK cells, and M1 macrophages were increased significantly, over all tested dual combinations. TUSC2 induces proliferation of NK cells through the upregulation of IL-15 signaling^8^. We showed that TUSC2 nanovesicles were taken up by NK cells and lineage-negative myeloid cells and induced IL-15 secretion, which led to NK cell proliferation. Increased NK activity was associated with generating Th1 immunity in TME. TUSC2 also downregulates the PD1/PD-L1 signaling in tumor cells^9^. CTL responses are associated with antitumor responses, which were reported by others and us in humanized mice^14,24,25^. Elevated CD8^+^ T cells displayed possible antigen-specific T cell responses, as demonstrated by their lack of reactivity towards non-tumor epithelial cells. The ratios of effector T cells over Tregs were also altered, favoring the effector T cell population. In addition, significantly higher levels of reconstituted human dendritic cells were found, including HLA-DR^+^ and CD11c^+^ DCs. The latter are potent antigen-presenting cells.

On the other hand, TUSC2 plus carboplatin and pembrolizumab reduced tumor infiltration by Tregs, MDSCs, TAMs, and M2 Macrophages significantly. M2-polarized TAMs drive multiple protumorigenic processes, including immunosuppression, metastasis, and tumor survival^26^. To our knowledge, this is the first study to characterize M1 and M2 macrophage polarization in the tumor microenvironment in humanized mice. These results indicate that TUSC2 combined with carboplatin and pembrolizumab affected many immune cell populations in the TME, significantly activating immune stimulatory while reducing immunosuppressive cell populations.

Complete eradication of KL tumors by the triple agent combination was also associated with increased central memory (CM) and effector memory (EM) T cells, significantly more than those in the carboplatin plus pembrolizumab treatment group. Antigen-experienced T cells undergo differentiation to memory T cells downregulating CD45RA expression to CD45RO, which persist for a longer time and provide antitumor immunity as compared with effector T cells. CD45RA antigens, identify functional characteristics of naive T lymphocytes, which lack HLA class II expression and do not respond to recall antigens, whereas their counterparts, CD45RO antigens, identify functional characteristics of memory T cells, which have high HLA class II expression and do respond to recall antigens^27^. Both CM and EM T cells provide strong antitumor immunity, which requires a very small amount of antigen exposure, compared to non-memory effector cells. A severe loss of T-memory cells during tumor progression has been reported, indicating a constant rivalry between cancer and T-memory cells^28^. Hence, it is conceivable that by increasing the memory population, the tumor burden can be reduced. Sorted CM and EM T cells from treated humanized tumors showed that TUSC2 addition to carboplatin and pembrolizumab correlated with a significant increase of (a) IFN-γ levels, after tumor antigens exposure, indicating antigen-specific immune responses of the tested memory T cells; and (b) tissue-resident T-memory cells (T_RM_). T_RM_, CD103^+^ CD8^+^ T cells are known to provide rapid protective immunity and are associated with a greater antitumor response. This suggests a specific role for these cells in producing potent antigen-specific immune responses by the triple combination^29–31^.

In conclusion, to be most effective, cancer therapy must take into account drug effects on the tumor microenvironment, which is dynamic and constantly shifting and which could be remodeled to enhance therapy. Collectively, our results demonstrate that TUSC2-stimulated immunity can sensitize resistant KL lung tumors to chemoimmunotherapy. The mechanisms of such synergy are multifaceted and are likely to involve other pathways, which remain to be investigated. The toxicity of carboplatin to immune cell populations can limit the extent of immune stimulation and lead to immunosuppression. Moreover, active expression and redistribution of PD-L1 to cancer cells may impair the therapeutic benefits of pembrolizumab. As carboplatin-induced tumor cell cytotoxicity and antitumor immune responses to carboplatin plus pembrolizumab wane, pro-tumor immune responses rebound. Addition of TUSC2, which downregulates PDL-1, could either induce de novo immunity or boost carboplatin plus pembrolizumab-stimulated immunity by providing alternative or complementary pathways to immune cell activation. This activity by TUSC2 induces strong antitumor immune responses, complementing and enhancing the immune stimulatory actions by carboplatin plus pembrolizumab. Importantly, the demonstrated therapeutic synergy of this triple agent combination supports the design of a clinical trial for the KL NSCLC subtype.

## Methods

### Cell line, cell culture, and maintenance

A549-luc human NSCLC cell line which carries KRAS/STK11 mutation was kindly provided by Dr. John Minna (The University of Texas Southwestern Medical Center, Dallas, Tx). A549-luc cells were cultured in RPMI-1640 medium supplemented with 10% heat-inactivated fetal bovine serum (GE Healthcare Life Sciences, HyClone Laboratories) and 1% penicillin–streptomycin (Thermo Fisher Scientific) at 37 °C with 0% CO_2_. KRASG12/CMT167-luc cells were kindly provided by Frank R. Jirik (University of Calgary). CMT167 cells were cultured in Dulbecco’s modified Eagle’s medium supplemented with 10% heat-inactivated fetal bovine serum (GE Healthcare Life Sciences, HyClone Laboratories) and 1% penicillin and streptomycin (Thermo Fisher Scientific). Both cell lines tested negative for mycoplasma before use in experiments. The cell lines were tested for mycoplasma routinely by ELISA in a core lab in MD Anderson Cancer Center. Both cell lines were also authenticated before the experiment by the core lab in The University of Texas MD Anderson Cancer Center.

### Mice used for humanization

NOD.Cg-*Prkdc*^*scid*^ *Il2rg*^*tm1Wjl*^/SzJ (NSG) mice were obtained from The Jackson Laboratory. This strain’s eligibility for engraftment of human hematopoietic cells has been described^32^. Female 3–4-week-old mice were used in these studies. Mice were housed in microisolator cages under specific pathogen-free conditions in a dedicated humanized mice room in the animal facility at The University of Texas MD Anderson Cancer Center. Mice were given autoclaved acidified water and fed a special diet (Uniprim diet). All animal use was conducted in accordance with the guidelines of the Animal Care and Use Committee of MD Anderson Cancer Center.

### Umbilical cord blood processing

Human umbilical cord blood units for research were obtained from MD Anderson Cord Blood Bank under an Institutional Review Board (IRB)-approved protocol (Lab04-0249). The cord blood bank collects umbilical cord blood through voluntary donations from mothers following informed consent under the institutional approved IRB protocol. Cord blood bank collects human cord blood on daily basis from several Houston-area hospitals like Memorial Hermann Hospital, St. Joseph Medical Center, and the Woman’s Hospital of Texas, etc. Fresh cord blood units were delivered to the research lab within 24 h of harvest, and the cord blood units were HLA typed immediately at MD Anderson HLA typing core facility. Cord blood was diluted to a ratio of 1:3 with phosphate-buffered saline, and mononuclear cells were isolated by using density-gradient centrifugation on Ficoll medium. The isolated mononuclear cells were directly used for CD34^+^ enrichment. For some experiments, CD34^+^ cells were also cultured for ex-vivo expansion, and the expanded cells were tagged with a NanoLuc lentivirus. The NanoLuc^+^ CD34^+^ cells were injected into mice for in vivo tracking.

### Humanized NSG mice

After mononuclear cells were separated from human umbilical cord blood, CD34^+^ HSPCs were isolated using a direct CD34^+^ MicroBead kit (Miltenyi Biotec). Three- to four-week-old NSG mice were irradiated with 200 cGy using a ^137^Cs gamma irradiator. Over 90% pure freshly isolated CD34^+^ HSPCs were injected intravenously, 24 h after irradiation, at a density of 1–2 × 10^5^ CD34^+^ cells/mouse. The engraftment levels of human CD45^+^ cells were determined in the peripheral blood, as early as 4 weeks post CD34 injection, by flow cytometric quantification, as well as other human immune populations. Mice with 25% human CD45^+^ cells were considered as humanized (Hu-NSG mice). In-depth analysis of bone marrow and spleen tissue for human immune cell subpopulations, including CD45^+^, CD3^+^, CD4^+^, and CD8^+^ T cells, B cells, NK cells, and lineage-negative cells, was performed using a 10-color flow cytometry panel at weeks 6 and 9 post CD34^+^ engraftment. Hu-NSG mice from different cord blood donors with different levels of engraftment were randomized into every treatment group in all of the experiments. All Hu-NSG mice were verified for humanization before tumor implantation.

### Human cell line xenografts development in mice

KRAS/LKB1 mutant A549-luc human NSCLC cell lines were cultured in RPMI-1640 medium supplemented with 10% heat-inactivated fetal bovine serum (GE Healthcare Life Sciences, HyClone Laboratories) and 1% penicillin–streptomycin (Thermo Fisher Scientific) at 37 °C with 5% CO_2_. Cell lines tested negative for mycoplasma before use. To generate experimental lung metastases, 1 × 10^6^ A549-luc cells were injected intravenously into NSG mice 6–8 weeks post CD34 engraftment. Tumor growth was measured by quantifying bioluminescence intensity with an IVIS small animal imaging system (IVIS 200; Caliper Lifesciences).

### TUSC2 nanovesicle formulation

*TUSC2*, previously known as *FUS1*, was encapsulated into DOTAP nanoparticles. The formulation is composed of 1, 2-bis(oleoyloxy)-3-(trimethylammonio)propane (DOTAP): cholesterol nanoparticles and a DNA plasmid expressing the TUSC2 tumor suppressor gene. The plasmid (PLJ 143/pKGB2/FUS 1) is 3968 bp and contains a kanamycin resistance gene, an origin of replication, and the human wild-type TUSC2 gene driven by a CMV promoter. The formulation is routinely manufactured in GMP facility following standard manufacturing protocols^10,33^. The GMP grade TUSC2 nanovesicles were used as a study agent in this study.

### Development and treatment of human A549 KRAS/LKB1 mutant xenografts in humanized mice

Seven days post A549 implantation in humanized mice, tumor growth and metastasis was confirmed by IVIS imaging. Then, animals were randomized into treatment and no-treatment groups on the basis of tumor intensity and donor HLA type. Eight to ten mice per group from multiple umbilical cord blood donors were used. The treatment groups were: untreated, TUSC2, carboplatin, pembrolizumab, TUSC2 + carboplatin, TUSC2 + pembrolizumab, carboplatin + pembrolizumab, and TUSC2 + carboplatin + pembrolizumab. TUSC2 (Genprex Inc) (25 ug/mouse) was injected intravenously every 3 days for three times; anti-PD1 agent pembrolizumab (Merck) or nivolumab (Bristol–Myers Squibb) (250 μg/mouse, intraperitoneally) every 3–4 days for 3 cycles; and carboplatin (Teva Pharmaceuticals) (10 mg/kg, orally). TUSC2 encapsulated nanovesicles were validated for their expression by transfecting into A549 cells before inoculation in humanized mice. A549 xenograft tumors were also generated in non-humanized NSG mice, which were treated alongside their humanized counterparts, as a control arm. Mice were monitored routinely for metastasis development with IVIS imaging. For immune analysis, mice from each treatment group were sacrificed 2 weeks post A459 inoculation, and organs were harvested for single-cell analysis. All animal experiments were carried out following approval by the MDACC institutional review board and were performed in accordance with the Guidelines for the Care and Use of Laboratory Animals published by the National Institutes of Health. All measurements quantifying experimental outcomes were blinded to the intervention.

### Immune analysis by flow cytometry

Erythrocytes in the peripheral blood were lysed with ACK lysis buffer (Fisher Scientific). Single-cell suspensions were prepared from fresh lung metastasis and spleen tissues using standard procedures. Several 10-color flow cytometry panels were used for immune profiling of both innate and adaptive immune populations in humanized mice and for evaluating immune response after treatment. Fluorochrome–conjugated monoclonal antibodies to the following human antigens were used: CD45-Alexa Fluor 700 (clone 2D1, HI30), CD45-phycoerythrin (PE; clone 2D1, HI30), CD3-PerCp/cy5.5 (clone HIT3a), CD19-PE-cyanine 7 (clone HIB19), CD8-allophycocyanin-cyanine 7 (clone RPA-T8, HIT8a), CD4-Pacific blue (clone OKT4), CD56-PE/BV510 (clone HCD56), CD69-FITC/APC/PE-Alexa Flour 610 (clone FN50; Thermo fisher), HLA-DR-PerCp/cy5.5 (clone LN3), CD33-PE (clone WM-53) (Thermo fisher), CD11b-PE-Cy7 (clone 1CRF-44) (Thermo fisher), Granzyme B-FITC (clone GB11), and IFN-γ-APC (clone 4 S.B3), CD103-Super bright 600 (Colne B-LY7; Thermo fisher), CD279 (PD-1)-Super Bright 702 (Clone J105; Thermo fisher), CCR7-FITC (Clone G043H7), CD45RA-PE (Clone HI100), CD25-APC (clone CD25-4E3), Lin-FITC (Biolegend), CD163-APC (clone ebioGH1/61; Thermo fisher), CD11c-Pacific blue (clone Bu15; Thermo fisher). A mouse CD45-FITC (clone 30-F11) antibody was used for gating out murine leukocytes. Most antibodies were purchased from Biolegend if otherwise mentioned. The dilutions of antibodies used in sample staining were followed according to the manufacturer’s protocol. All samples were run on Attune NxT flow cytometer (Thermo Fisher), and data were analyzed by Flow Jo and Kaluza software packages.

### Functional assay for T cells and sorted memory T cells harvested from humanized mice

After treatment, mice were euthanized, and their spleens were harvested. Splenocytes were co-cultured with heat-killed A549 cells (30 min in a 50 °C water bath) or PMA (Phorbol 12-myristate 13-acetate; Sigma-Aldrich) and ionomycin (Sigma-Aldrich) for 4 h in the presence of a protein transport inhibitor-containing brefeldin A (GolgiPlug, BD Biosciences). Intracellular staining for IFN-γ (Biolegend) and Granzyme B (Biolegend) was performed according to the manufacturer’s protocol (BD Biosciences). Percentage of IFN-γ expressing CD3^+^, CD4^+^, CD8^+^, effector memory, and central memory T cells were determined by FACS. Human bronchial epithelial cells (HBECs) were used as an HLA-matched control for A549 cells (HLA-A*02).

One week after treatment, mice were sacrificed, spleens were harvested, and single-cell suspensions were prepared freshly. In each treatment group, at least 3–5 mice splenocytes were pooled and stained for 30 min with human CD45, CD3, CCR7, and CD45RA antibodies. Immediately after staining, memory T cells were sorted by Aria II sorter (BD Bioscience) as EM/CM^+^ T cells, EM/CM^−^ T cell fractions by the following gating: EM/CM^+^ T cells (CD45^+^ > CD3^+^ > CD45RA^−^CCR7^+/−^) and EM/CM^−^ T cells (CD45^+^ > CD3^+^ > CD45RA^+^CCR7^+/−^). Sorted memory T cells were co-cultured with heat-killed A549 or treated with PMA (Phorbol 12-myristate 13-acetate; Sigma-Aldrich) and ionomycin (Sigma-Aldrich) for 4 h in the presence of a protein transport inhibitor-containing brefeldin A (GolgiPlug, BD Biosciences). Intracellular staining for IFN-γ (Biolegend) was performed according to the manufacturer’s protocol (BD Biosciences).

### Statistics and reproducibility

Statistical analyses were performed with GraphPad Prism 7 software. Tumor intensity change per time point was calculated as a relative level of tumor intensity change from baseline. Two-way ANOVA with the interaction of treatment group and time point was performed to compare the difference of tumor intensity changes from baseline between each pair of the treatment groups at each time point. Means ± standard errors of the mean are shown in all graphs. Differences of *P* < 0.05, *P* < 0.01, and *P* < 0.001 were considered statistically significant. Statistical analysis of flow cytometry data was done by generalized linear regression models to compare the data among the different treatment groups using PROC GENMOD in SAS version 9.2. Both nom *P*-values and multiple testing adjusted *P*-values were reported. For the Forest plot, the data were analyzed based on the ANOVA models with main effects of treatment and cell type variables and the post-hoc analysis using Tukey’s Honest Significant Difference (HSD) method. Ninety percent (90%) family-wise confidence levels and adjusted *p*-values on the differences between the means of the levels of each factor were computed.

### Reporting summary

Further information on research design is available in the Nature Research Reporting Summary linked to this article.

## Supplementary information

Supplementary Information

Description of Additional Supplementary Files

Supplementary Data 1

Reporting Summary

## Data Availability

All data generated or analyzed during this study are included in this published article. The source data underlying most graphs and charts used in this manuscript are provided as a Supplementary Data File (excel). Request for any source data or materials that are not provided should be made to the corresponding author.
