# Supplementary material for: TUSC2 immunogene enhances efficacy of chemo-immuno combination on KRAS/LKB1 mutant NSCLC in humanized mouse model
Source: Commun Biol. 2022 Feb 24;5:167. doi: 10.1038/s42003-022-03103-7 (PMC8873264; doi:10.1038/s42003-022-03103-7)
Supplement: Supplementary file 1 — Supplementary Information [file 42003_2022_3103_MOESM1_ESM.pdf]

TUSC2 immunogene enhances efficacy of chemo-immuno combination on KRAS/LKB1 mutant NSCLC in humanized mouse model

Ismail M. Meraz[1*], Mourad Majidi[1], RuPing Shao[1], Feng Meng[1], Min Jin Ha[2], Elizabeth Shpall[3], Jack A. Roth[1, 4]

[1]Department of Thoracic and Cardiovascular Surgery, The University of Texas MD Anderson Cancer Center, Houston, Texas, USA.

[2]Department of Biostatistics, The University of Texas MD Anderson Cancer Center, Houston, Texas, USA.

[3]Department of Stem Cell Transplantation, The University of Texas MD Anderson Cancer Center, Houston, Texas, USA.

[4]Department of Thoracic Medical Oncology, The University of Texas MD Anderson Cancer Center, Houston, Texas, USA.

Supplementary Information File

Supplementary Figure 1

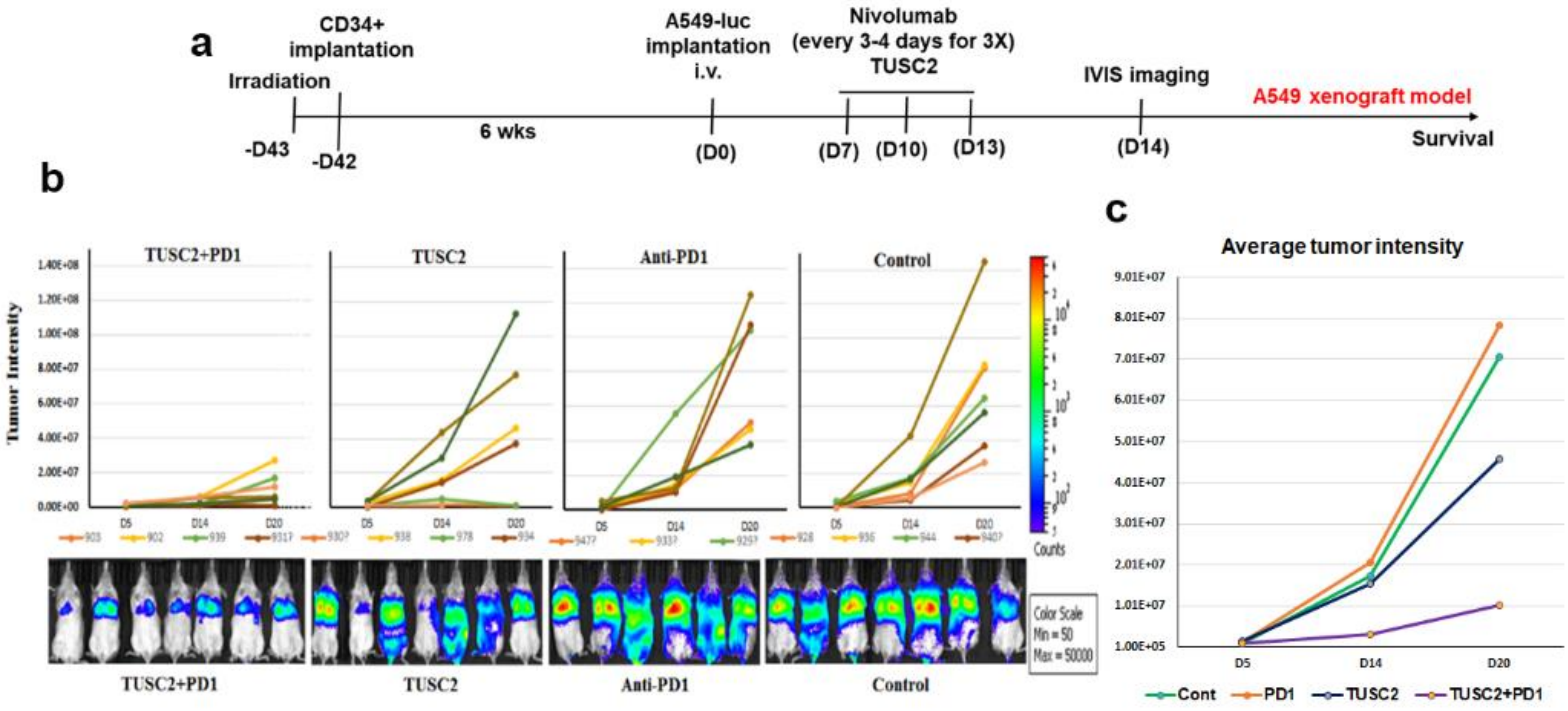

Supplement 1. Antitumor immune effect of TUSC2+nivolumab on lung metastases in a humanized mouse model. a) treatment strategy, b) antitumor response of TUSC2 and nivolumab single treatment and combination. Each line shows the individual mouse response to the treatment (left panel). C) the average tumor burden determined by quantitation of the tumor intensity.

Supplementary Figure 2

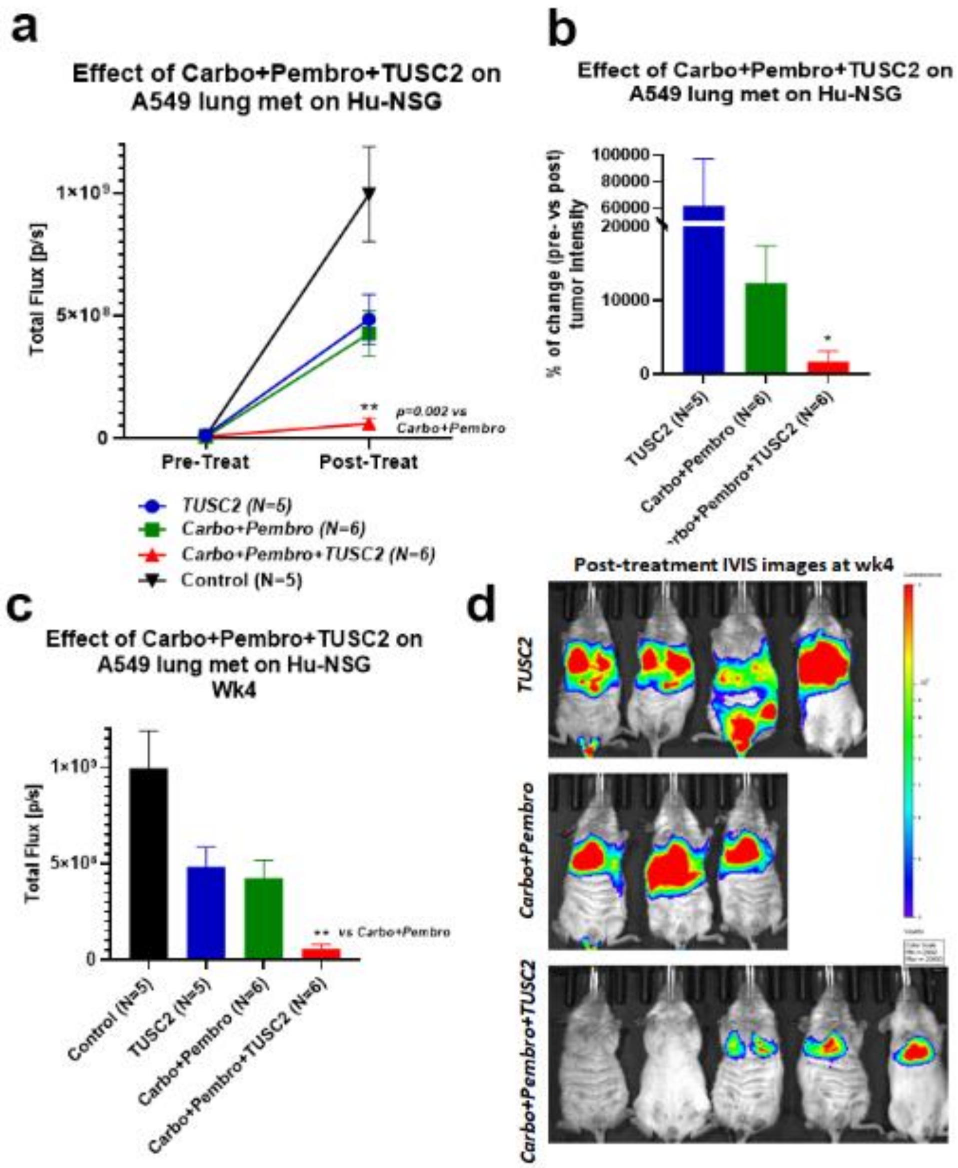

Supplement 2. Antitumor activity of triple agent combination on KL-mutant lung metastases in a humanized mouse model. This is one experiment of three independent experiments a) tumor intensity measured by IVIS imaging following different treatments. b) percentage change of tumor burden between pre- and post-treatment. c) tumor burden status at wk 4 after treatment. d) IVIS images of post-treated mice at wk 4.

Supplementary Figure 3

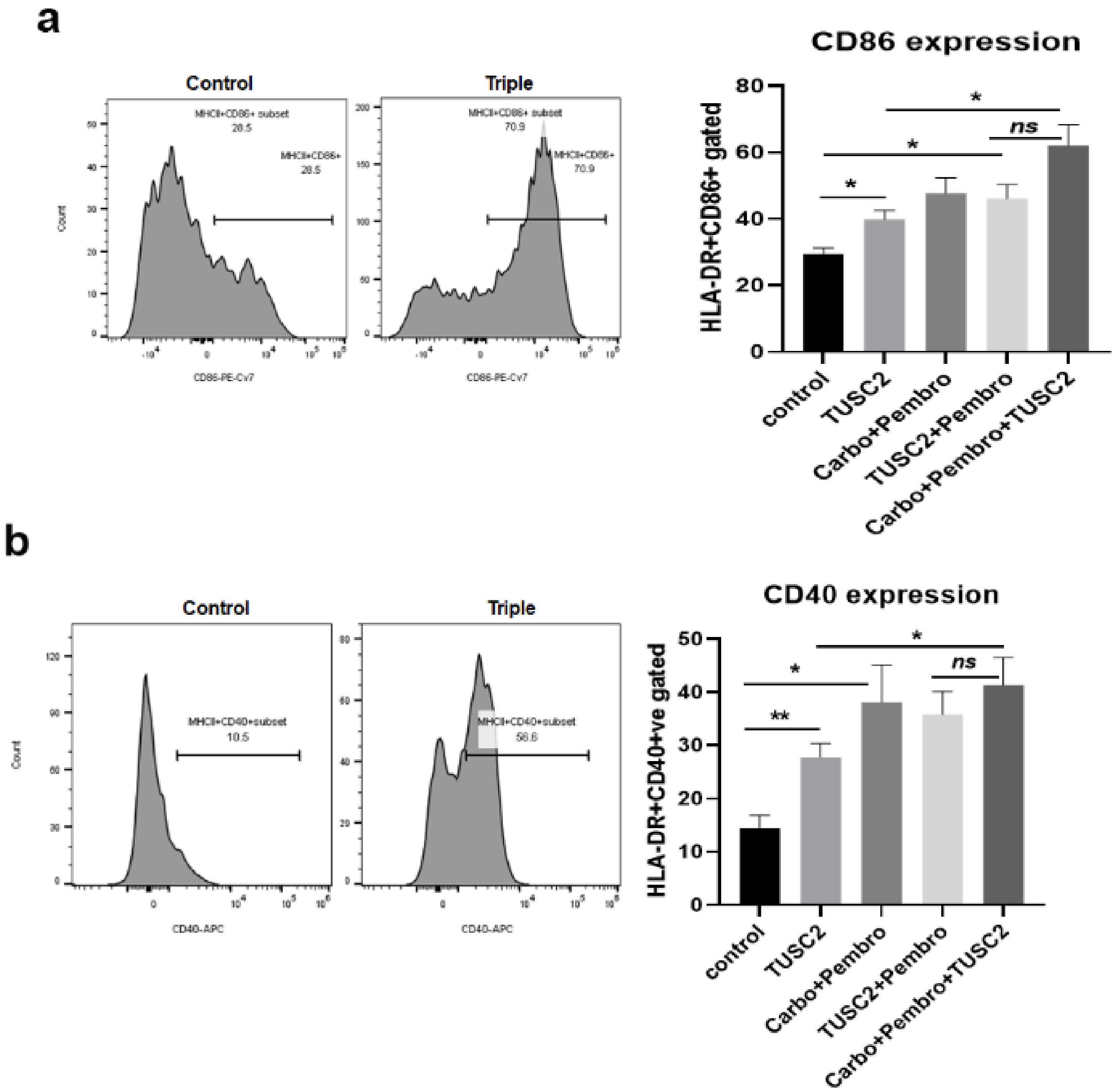

Supplement 3. Maturation of Dendritic cells in humanized mice. Tumor bearing humanized mice were treated with different regimens and infiltrating DC were analyzed for maturation marker analysis by flow cytometry. a) CD86 expression on HLA-DR+ DC infiltrated into TME. b) CD40 expression on HLA-DR+ DC infiltrated into TME.

Supplementary Figure 4

	Difference (90% Confidence level)	p−value
TUSC2 vs. Control	1.7 (−3.8,7.2)	0.92632
Carbo+Pembro vs. Control	0.1 (−5.4,5.6)	1
TUSC2+Pembro vs. Control	5 (−0.5,10.6)	0.15439
Carbo+Pembro+TUSC2 vs. Control	7.2 (1.6,12.7)	0.01911
Carbo+Pembro vs. TUSC2	−1.6 (−7.1,3.9)	0.94163
TUSC2+Pembro vs. TUSC2	3.3 (−2.2,8.9)	0.52515
Carbo+Pembro+TUSC2 vs. TUSC2	5.4 (−0.1,11)	0.10787
TUSC2+Pembro vs. Carbo+Pembro	4.9 (−0.6,10.5)	0.17019
Carbo+Pembro+TUSC2 vs. Carbo+Pembro	7 (1.5,12.6)	0.0216
Carbo+Pembro+TUSC2 vs. TUSC2+Pembro	2.1 (−3.4,7.6)	0.85521
EM/CM+ vs. EM/CM−	5.2 (2.9,7.5)	0.00072

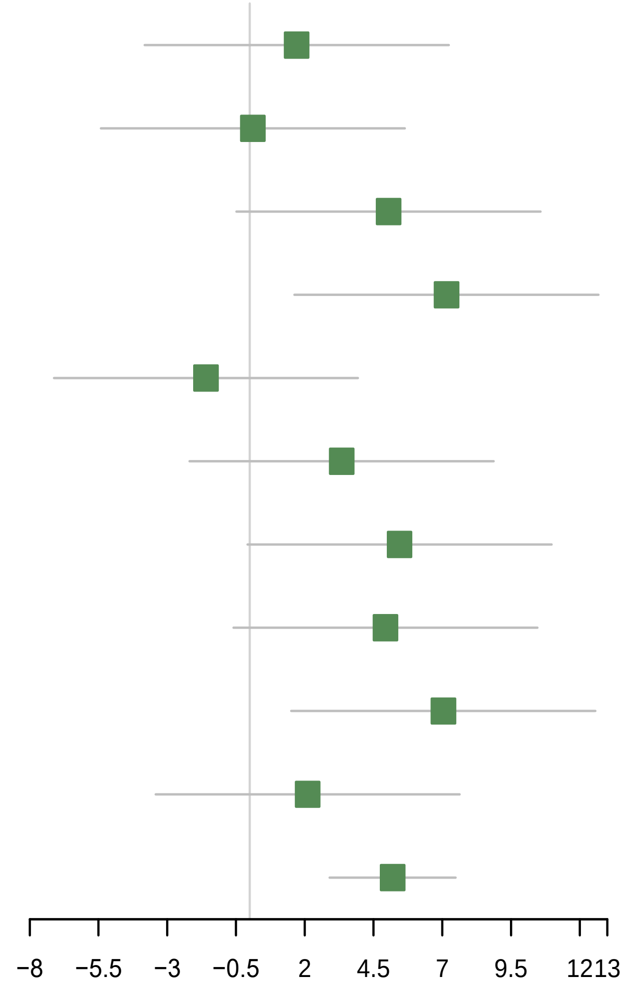

Supplement 4. IFN-γ responses from sorted Effector/Central memory T cells harvested from tumor bearing treated humanized mice. Tumor bearing humanized mice were treated with different treatment regimens followed by harvesting the T cells. Then Effector/Central memory T cells were sorted by cell sorter and separated EM/CM+ T cells from EM/CM- T cells. IFN-γ responses were evaluated shown in figure 7F. The data were statistically analyzed for the Forest plot for difference in means, the 90% family-wise confidence level, and adjusted p-value.

Results: The mean of Carbo+Pembro+TUSC2 was significantly different from those of control and carbo+pembro treatment groups with multiplicity adjusted p-values of 0.0191 and 0.0216, respectively. EM/CM+ and EM/CM- showed a significant difference in means for the dataset with the adjusted p-values of 0.00072.

Supplementary Figure 5

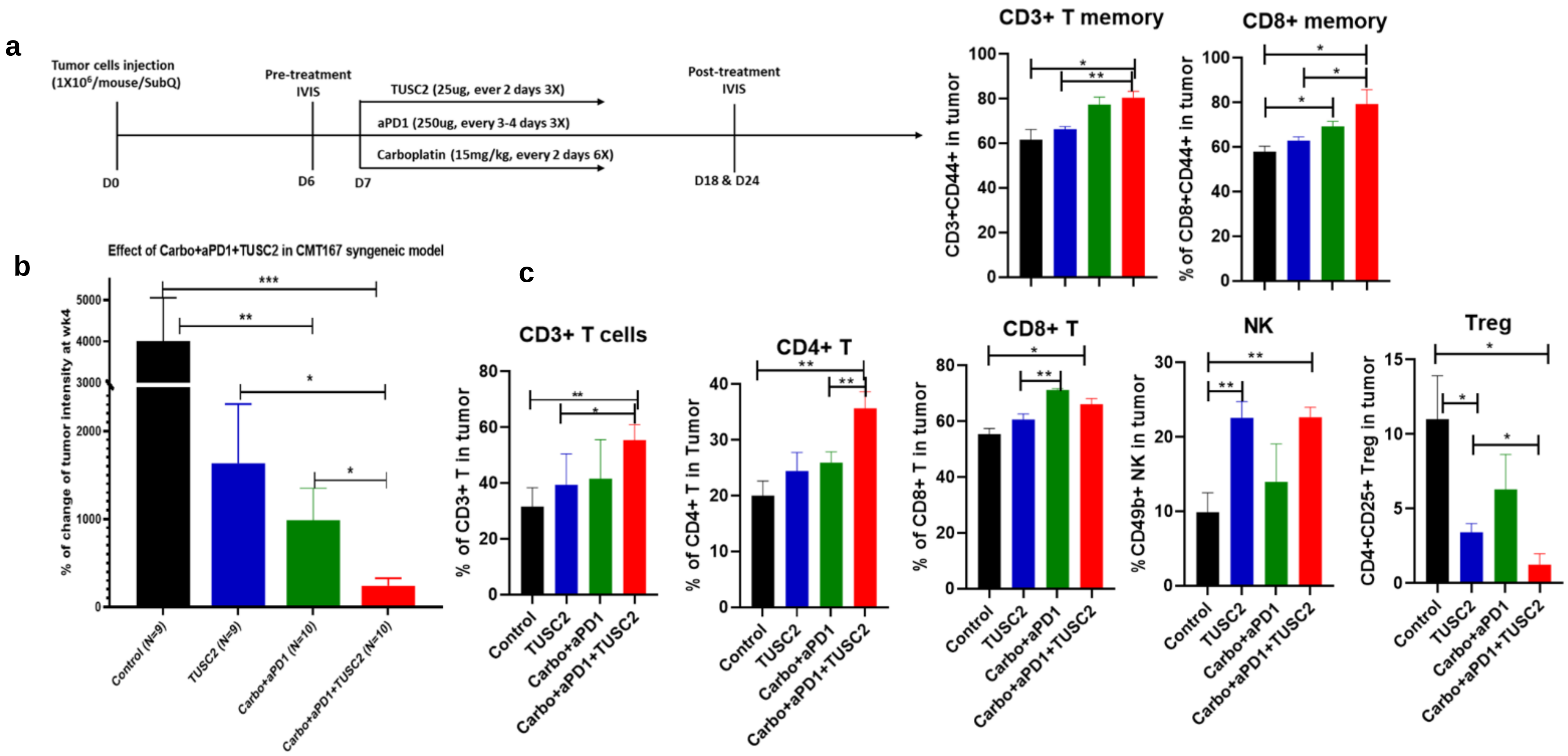

Supplement 5. Antitumor immune response of Carboplatin + aPD1 + TUSC2 against CMT167 tumors in syngeneic mouse model. a) experimental strategy, b) antitumor effect shown as percentage of tumor intensity change before and after treatment, c) Effect of triple treatment on immune cells in tumor microenvironment.
