# Supplementary material for: TUSC2 immunogene enhances efficacy of chemo-immuno combination on KRAS/LKB1 mutant NSCLC in humanized mouse model
Source: Commun Biol. 2022 Feb 24;5:167. doi: 10.1038/s42003-022-03103-7 (PMC8873264; doi:10.1038/s42003-022-03103-7)
Supplement: Supplementary file 2 — Description of Additional Supplementary Files [file 42003_2022_3103_MOESM2_ESM.pdf]

Description of Additional Supplementary Files

File name: Supplementary Data 1
Description: Source data underlying most graphs and charts used in this manuscript.
