# Supplementary material for: TUSC2 immunogene enhances efficacy of chemo-immuno combination on KRAS/LKB1 mutant NSCLC in humanized mouse model
Source: Commun Biol. 2022 Feb 24;5:167. doi: 10.1038/s42003-022-03103-7 (PMC8873264; doi:10.1038/s42003-022-03103-7)
Supplement: Supplementary file 4 — Reporting Summary [file 42003_2022_3103_MOESM4_ESM.pdf]

nature portfolio

Corresponding author(s): ISMAIL M MERAZ

Last updated by author(s): Aug 30, 2021

Reporting Summary

Nature Portfolio wishes to improve the reproducibility of the work that we publish. This form provides structure for consistency and transparency in reporting. For further information on Nature Portfolio policies, see our Editorial Policies and the Editorial Policy Checklist.

Statistics

For all statistical analyses, confirm that the following items are present in the figure legend, table legend, main text, or Methods section.

n/a	Confirmed	
☐	☒	The exact sample size (n) for each experimental group/condition, given as a discrete number and unit of measurement
☐	☒	A statement on whether measurements were taken from distinct samples or whether the same sample was measured repeatedly
☐	☒	The statistical test(s) used AND whether they are one- or two-sided<br>*Only common tests should be described solely by name; describe more complex techniques in the Methods section.*
☒	☐	A description of all covariates tested
☐	☒	A description of any assumptions or corrections, such as tests of normality and adjustment for multiple comparisons
☐	☒	A full description of the statistical parameters including central tendency (e.g. means) or other basic estimates (e.g. regression coefficient) AND variation (e.g. standard deviation) or associated estimates of uncertainty (e.g. confidence intervals)
☐	☒	For null hypothesis testing, the test statistic (e.g. F, t, r) with confidence intervals, effect sizes, degrees of freedom and P value noted<br>*Give P values as exact values whenever suitable.*
☒	☐	For Bayesian analysis, information on the choice of priors and Markov chain Monte Carlo settings
☒	☐	For hierarchical and complex designs, identification of the appropriate level for tests and full reporting of outcomes
☒	☐	Estimates of effect sizes (e.g. Cohen's d, Pearson's r), indicating how they were calculated

Our web collection on statistics for biologists contains articles on many of the points above.

Software and code

Policy information about availability of computer code

Data collection	No Software was used
Data analysis	No Software was used

For manuscripts utilizing custom algorithms or software that are central to the research but not yet described in published literature, software must be made available to editors and reviewers. We strongly encourage code deposition in a community repository (e.g. GitHub). See the Nature Portfolio guidelines for submitting code & software for further information.

Data

Policy information about availability of data

All manuscripts must include a data availability statement. This statement should provide the following information, where applicable:

- Accession codes, unique identifiers, or web links for publicly available datasets
- A description of any restrictions on data availability
- For clinical datasets or third party data, please ensure that the statement adheres to our policy

All relevant or raw are available from the corresponding author upon request. All other data supporting the findings of this study are available within the article or in supplement files.

Field-specific reporting

Please select the one below that is the best fit for your research. If you are not sure, read the appropriate sections before making your selection.

☒ Life sciences ☐ Behavioural & social sciences ☐ Ecological, evolutionary & environmental sciences

For a reference copy of the document with all sections, see nature.com/documents/nr-reporting-summary-flat.pdf

Life sciences study design

All studies must disclose on these points even when the disclosure is negative.

Sample size	All animal experiments, N=5-8 humanized mice/group was used unless otherwise mentioned in the results, figures or methods. Same number of animals were also used for all control groups.
Data exclusions	No data was excluded from the analysis
Replication	All experiments were repeated (otherwise mentioned) to make sure the reproducibility.
Randomization	All humanized mice were randomly assigned to the treatment groups. The humanized mice generated from single donor cord blood stem cells were even distributed into all treatment groups to make sure all treatment groups have humanized mice generated from multiple donors.
Blinding	Randomized were blindly allocated to treatment groups.

Reporting for specific materials, systems and methods

We require information from authors about some types of materials, experimental systems and methods used in many studies. Here, indicate whether each material, system or method listed is relevant to your study. If you are not sure if a list item applies to your research, read the appropriate section before selecting a response.

Materials & experimental systems

n/a	Involved in the study	
☐	☒	Antibodies
☐	☒	Eukaryotic cell lines
☒	☐	Palaeontology and archaeology
☐	☒	Animals and other organisms
☒	☐	Human research participants
☒	☐	Clinical data
☒	☐	Dual use research of concern

Methods

n/a	Involved in the study	
☒	☐	ChIP-seq
☐	☒	Flow cytometry
☒	☐	MRI-based neuroimaging

Antibodies

Antibodies used	Materials and Methods have all antibodies information such as cat#, clone, florescence conjugation etc used in flow cytometry
Validation	All Antibodies used in this study were validated by as well as the vendor.

Eukaryotic cell lines

Policy information about cell lines

Cell line source(s)	Human NSCLC cell line A549-luc was obtained from Dr, John Minna from University of Texas Southwestern University, TX.
Authentication	The cell line was authenticated by MD Anderson Cancer Center Core facility by STR method.
Mycoplasma contamination	The cells used in this study were tested negative for mycoplasma and tests were done by MD Anderson Cancer Center Core facility.
Commonly misidentified lines (See ICLAC register)	*Name any commonly misidentified cell lines used in the study and provide a rationale for their use.*

Animals and other organisms

Policy information about studies involving animals; ARRIVE guidelines recommended for reporting animal research

Laboratory animals	NOD.Cg-Prkdcscid Il2rgtm1Wjl/SzJ (NSG) mice, 3-5 wks old mice, Female for humanization.
Wild animals	This study did not invovle wild animals
Field-collected samples	N/A
Ethics oversight	All animal use was conducted in accordance with the guidelines of the Animal Care and Use Committee of MD Anderson Cancer Center.

Note that full information on the approval of the study protocol must also be provided in the manuscript.

Flow Cytometry

Plots

Confirm that:

☒ The axis labels state the marker and fluorochrome used (e.g. CD4-FITC).

☒ The axis scales are clearly visible. Include numbers along axes only for bottom left plot of group (a 'group' is an analysis of identical markers).

☒ All plots are contour plots with outliers or pseudocolor plots.

☒ A numerical value for number of cells or percentage (with statistics) is provided.

Methodology

Sample preparation	Single cell suspension was prepared from tumor tissues by using enzymatic digestion followed by filtration through 70nm filter and cells viability was tested.
Instrument	Attune NxT Flow Cytometer was used.
Software	Flow Jo was used for analysis
Cell population abundance	Around 100K events were collected in a SSC vs FSC scatter plot gated for lymphocyes in a single cell suspension sample prepared from tumor tissues. So that in the sub-gated population (target sub population) had at least 10K cells in the final analysis.
Gating strategy	All panels were compensated and validated before running any samples. Most gating strategies were mentioned and shown in the figures.

☐ Tick this box to confirm that a figure exemplifying the gating strategy is provided in the Supplementary Information.
